# Chemotherapy-Induced Alopecia Beyond Cytotoxicity: Hair Follicle Immune Privilege Collapse and JAK-STAT Signaling

**DOI:** 10.3390/ijms27104173

**Published:** 2026-05-07

**Authors:** Pin-Chi Wang, Sebastian Yu

**Affiliations:** 1 Department of General Medicine, Shuang Ho Hospital, Taipei Medical University, New Taipei City 235041, Taiwan; 25231@s.tmu.edu.tw; 2 Department of Dermatology, Kaohsiung Chang Gung Memorial Hospital and Chang Gung University College of Medicine, Kaohsiung 833401, Taiwan; 3 Master of Public Health Program, College of Public Health, National Taiwan University, Taipei 100025, Taiwan; 4 Department of Dermatology, School of Medicine, College of Medicine, Kaohsiung Medical University, Kaohsiung 807378, Taiwan; 5 Department of Dermatology, Kaohsiung Medical University Hospital, Kaohsiung 807377, Taiwan

**Keywords:** chemotherapy-induced alopecia, hair follicle, immune privilege collapse, JAK-STAT signaling, JAK inhibitors, immunodermatology, molecular pathogenesis

## Abstract

Chemotherapy-induced alopecia (CIA) is a distressing side effect of cancer treatment with limited effective therapeutic options. While CIA has traditionally been attributed to direct p53-mediated cytotoxicity against rapidly proliferating keratinocytes in hair bulbs, emerging evidence suggests a more complex pathogenesis that involves immune-mediated mechanisms analogous to those in alopecia areata (AA). This review synthesizes current literature and proposes that CIA may be fundamentally driven by the collapse of hair follicle (HF) immune privilege (IP). We explore how chemotherapy-induced DNA damage and psychophysiological stress converge to trigger inflammation, characterized by interferon-γ (IFN-γ)-driven pathways, oxidative stress, and neuroimmune dysregulation. We highlight histopathological, genetic, and clinical overlaps between CIA and AA, particularly regarding the shared involvement of the Janus Kinase-Signal Transducer and Activator of Transcription (JAK-STAT) signaling pathway. Consequently, Janus kinase (JAK) inhibitors (JAKi) are evaluated as potential therapeutic agents for CIA. However, the application of JAKi in oncologic populations requires scrutiny regarding potential immunosuppression and risks of malignancies. Finally, we discuss the context-dependent roles of cytokines involved in the HF-IP collapse pathway, such as interleukin (IL)-15 and IL-1, in HF homeostasis versus inflammation, and we outline future research directions for targeted and safe therapeutic strategies that mitigate CIA without compromising cancer treatment outcomes.

## 1. Introduction

Chemotherapy-induced alopecia (CIA) is the most frequently encountered hair disorder in the oncology setting, with an estimated incidence of approximately 65% among patients receiving cytotoxic systemic therapies [1]. This distressing side effect of cancer treatment has prompted numerous therapeutic investigations that have yielded inconsistent results. Currently, scalp cooling represents the most evidence-based preventive approach, yet its substantial cost limits its accessibility [2,3,4].

Traditional paradigms of the pathogenesis of CIA have primarily attributed hair loss to the direct cytotoxic effects of chemotherapeutic agents on highly proliferative hair bulb keratinocytes and the follicular pigmentary system [5,6], and in severe cases, also hair follicle (HF) stem cells (HFSCs) [7,8]. Proposed mechanisms responsible for CIA involve the activation of multiple apoptotic pathways, including but not limited to p53 and Fas [9,10], and the suppression of Sonic hedgehog (Shh) signaling, which ultimately leads to caspase-mediated massive HF apoptosis [11,12].

Beyond DNA damage-induced apoptotic pathways, a comprehensive review of CIA literature reveals a potential convergence with alopecia areata (AA) through similar upstream pathways culminating in inflammation-associated immune privilege (IP) collapse. Paus et al. recently proposed a paradigm-shifting concept, stating that “AA does not necessarily represent a single disease entity, but rather it encompasses a stereotypic HF response pattern.” Once HF-immune privilege (IP) fails to prevent interferon-γ (IFN-γ)-induced activation of natural killer (NK) cells and cytotoxic (CD8^+^) T cells, immune-mediated hair disorders can occur even in the absence of antigen-dependent autoimmunity [13].

The potency of IFN-γ is particularly evident in that purified CD8^+^ T cells or NK cells capable of secreting IFN-γ alone suffice to induce an AA phenotype in HFs [14]. This broader view suggests the hypothesis that immune-mediated HF alterations in CIA, driven by IFN-γ secondary to DNA damage-associated oxidative stress [15] and substance P (SP) that arises from cancer-related psychological stress, may trigger HF-IP collapse [16].

In a recent publication, Guan et al. [17] demonstrated that the Janus kinase (JAK) inhibitor (JAKi) tofacitinib effectively treats CIA in murine models. The established mechanism of tofacitinib further involves the modulation of CD8^+^ T-cell-related pathways, including the upstream IFN-γ and interleukin (IL)-15 signaling cascades, which play critical roles in the pathogenesis of autoimmune disease [18]. The demonstrated efficacy of JAKi in CIA, together with previous findings using immunosuppressants or immunomodulators, suggests the potential involvement of immune mechanisms analogous to those observed in AA.

This review aims to synthesize current understanding of IP collapse in CIA, integrate accumulating evidence supporting an immune-mediated contribution to its pathogenesis, and propose future directions for therapeutic research development.

## 2. HF-IP Maintenance and Inflammation-Associated IP Collapse

### 2.1. Maintenance and Counter-Regulatory Mechanisms of HF-IP

Anagen HFs function as immune-privileged micro-organs due to their rapid proliferative nature and tendency to display autoantigens. Multi-tiered strategies are employed to prevent excessive inflammation-induced IP damage and subsequent hair loss.

At the molecular and cellular levels, HF-IP is maintained through the active suppression of immunogenic signals, marked by minimal expression of major histocompatibility complex (MHC) class I and II molecules, human leukocyte antigens (HLA), including HLA-A, HLA-B, HLA-C, β2-microglobulin, and a sparse population of antigen-presenting cells (APCs) around HF [19,20]. Simultaneously, the HF microenvironment is enriched with immunosuppressive mediators, including α-melanocyte-stimulating hormone (α-MSH), indoleamine 2,3-dioxygenase (IDO), migration inhibitory factor (MIF), CD200, and HLA-E, collectively inhibiting the infiltration and activity of cytotoxic immune cells [21,22,23,24,25,26,27,28,29].

This privilege is spatially graded. The hair bulge, which harbors HFSCs in each HF, boasts a more robust and specialized IP niche than the hair bulb. This enhanced state is underpinned by attenuated MHC class I and II expression and a substantial enrichment of IDO and MIF relative to the bulb region [30]. Key immunomodulators, like CD200 and HLA-E, are almost exclusively restricted to the hair bulge region [31]. This superior IP is manifested by a 10-fold higher resistance to IFN-γ-induced collapse than that necessary to collapse hair bulb IP [32], explaining the relative preservation of HFSCs in reversible CIA. However, the HF immune-privileged state is not static. Under inflammatory stress, HFs engage counter-regulatory mechanisms, primarily through IL-10 and TGF-β, to stabilize the immunosuppressive microenvironment and repair IFN-γ-induced damage [33,34,35]. The collapse of HF-IP occurs when pro-inflammatory triggers such as IFN-γ, tumor necrosis factor-α (TNF-α), ILs, or oxidants overwhelm these compensatory mechanisms and lead to hair loss [36].

### 2.2. Inflammation-Associated HF-IP Collapse and Perifollicular Remodeling

#### 2.2.1. The IFN-γ Axis and HF-IP Collapse

The core pathogenic mechanism underlying immune-mediated hair loss disorders is driven by IFN-γ–induced Th1-mediated inflammation. Often upregulated in response to physiological stress, IFN-γ and its downstream chemokines, C-X-C motif chemokine (CXCL) 9 and CXCL10, constitute the signature cytokines of AA and are elevated in the serum and the localized lesions of affected patients [37,38,39]. Lesional overexpression of CXCL10 facilitates the recruitment of CD8^+^ T cells displaying heightened migratory responsiveness to CXCL10 [40]. These inflammatory cues precipitate aberrant MHC expression and IP collapse, prompting the expansion of CD8^+^ T cell populations and immune attack against hair bulb keratinocytes and melanocytes [41,42].

TNF-α acts as another pivotal upstream inflammatory mediator of immune-mediated hair disorders [43]. Along with its role in antigen-specific T cell-mediated immunity [44], TNF-α sensitizes cells to IFN-γ [45] and promotes the apoptosis of hair bulb keratinocytes [46,47,48]. TNF-α also induces the generation of reactive oxygen species (ROS) in HF melanocytes and compromises their survival and melanin synthesis capacity [49,50].

#### 2.2.2. Melanocyte and Mast Cell Dysregulation in the Perifollicular Niche During HF-IP Collapse

The survival and normal function of the melanocytes within anagen hair bulbs are also disrupted in the context of hair loss [51]. HF melanocytes are referred to as immunocompetent cells that play indispensable roles in immune regulation [52,53]. Upon stimulation by antigens, ROS, or IFN-γ, they release pro-inflammatory mediators including TNF-α, IL-1β, IL-6, and IL-8 [30,54,55,56,57]. Additionally, functional melanocytes producing melanin and expressing strong immunogenic antigens render pigmented HFs more vulnerable to CD8^+^ T cell-dependent immune attack induced by IFN-γ [50,58,59,60,61,62,63]. This may explain the predilection for pigmented hair loss observed in autoimmune AA [8].

Mast cells express IL-6 and respond to oxidative or psychological stress [64]. Depending on the cytokine milieu, such as interactions with CD8^+^ T cells and local concentrations of TGF-β and IL-10, perifollicular mast cells can switch to a pro-inflammatory phenotype [33]. This transition triggers the release of IL-6, a key mediator that facilitates autoantigen presentation to CD8^+^ T cells [65,66,67]. Parallel to this, IL-6 exerts a direct inhibitory effect on hair shaft elongation and keratinocyte proliferation, promoting premature catagen onset and hair loss [68,69,70].

The convergence of these cytokine axes and cell-specific vulnerabilities culminates in profound inflammatory remodeling. A defining histopathological characteristic of immune-mediated hair loss disorders is the presence of peribulbar inflammation and inflammatory cell exocytosis that affects hair bulb keratinocytes and melanocytes [71]. IFN-γ stimulation elicits perifollicular infiltration by immune cells that should be absent or minimal under IP conditions, including NK cells, CD8^+^ T cells, dendritic cells, and Langerhans cells [72,73]. Among the peribulbar infiltrates, NK cells and CD8^+^ T cells can also produce IL-17, which has been recently hypothesized to play a more important role in the pathogenesis of chronic autoimmune alopecia than IFN-γ [74,75,76].

These infiltrates attack anagen follicles and induce hair bulb apoptosis, further amplifying the secretion of Th1-associated chemokines, particularly CXCL10 [77]. This creates a self-perpetuating inflammatory loop where intrafollicular stress signaling and perifollicular immune cell recruitment continuously reinforce one another, fostering the inflammatory state and the failure of hair growth.

While these local inflammatory loops sustain the destruction of HF, they may not function in isolation. Psychophysiological stress is known to serve as a potent trigger for HF-IP collapse through the intrafollicular stress axis. We will explore how stress-induced neurogenic inflammation exacerbates HF-IP collapse in the following section.

### 2.3. Stress-Induced Neurogenic Inflammation and the Intrafollicular Stress Axis

The intrafollicular stress axis has been demonstrated to exist and mirror the systemic hypothalamic–pituitary–adrenal (HPA) axis, operating through the following cascade: corticotropin-releasing hormone (CRH) → pro-opiomelanocortin (POMC)-derived peptides including adrenocorticotropic hormone (ACTH) and α-MSH → cortisol. Psychophysiological stress can activate this cascade, elevating ACTH, α-MSH, and cortisol concentrations in the follicular microenvironment [55,56]. Strong immunostaining for these stress mediators has been observed in the epidermis of AA lesional skin [78]. This is accompanied by increased populations of mast cells and Langerhans cells, and the downstream effector IL-6, which serves as a crucial intermediary linking the HF stress axis to inflammation-driven HF-IP collapse [79].

The biological impact of this axis is mediated by several parallel pathways that disrupt HF homeostasis. CRH, the most upstream factor in the HPA axis, directly induces catagen, suppresses hair shaft growth, and modulates keratinocyte proliferation and apoptosis [80,81]. CRH and its downstream effector ACTH stimulate the production of stem cell factor (SCF) that drives the maturation and activation of perifollicular mast cells [82,83].

Cortisol, the terminal product of the intrafollicular stress axis, promotes the release of SP from keratinocytes [84]. SP interacts with mast cells via neurokinin-1 (NK-1) receptors to enhance the release of TNF-α and upregulate nerve growth factor (NGF) [85,86,87]. NGF is recognized as a critical mediator of stress responses [88] and participates in inducing mast cell activation and degranulation [89], which triggers perifollicular infiltration and the premature entry into catagen [83,90,91,92]. Additionally, stress-induced catecholamines also trigger lymphocytosis and are associated with hair loss [31,93].

Despite the above-mentioned destructive signals, the intrafollicular stress axis also harbors counter-regulatory mechanisms aimed at preserving IP. For instance, α-MSH has been shown to restore HF-IP collapse and display potent immunosuppressive properties [34,94]. Moreover, Calcitonin gene-related peptide (CGRP) regulated by stress through glucocorticoid and catecholamine signaling, counteracts IFN-γ-induced IP collapse by inhibiting MHC class I and II expression, reducing mast cell degranulation, and suppressing Langerhans cell activity [95,96,97]. Recent studies have shown that CGRP reduction in HFs or CGRP antagonists is associated with immune-mediated hair loss [98,99], supporting the protective role of CGRP in HF-IP.

In summary, physiological stress contributes to HF-IP collapse and hair loss by stimulating the intrafollicular stress axis, inducing skin mast cell degranulation, and generating neurogenic inflammation [92,100,101].

## 3. Hypothesized DNA Damage and Stress-Induced HF-IP Collapse

CIA is generally classified as non-scarring alopecia, characterized by alterations in follicular cycle homeostasis [102]. Chemotherapy-induced hair cycle abnormalities predominantly present as anagen effluvium, defined by shedding fully pigmented hair shafts while HFs remain in the growth phase of the hair cycle. Not only HF tissue damage caused by systemic chemotherapy, but also localized stressors, including ischemia and inflammatory responses following medical procedures, can disrupt follicular integrity and cause acute anagen cessation [103]. This phenomenon highlights the universal sensitivity of HF to exogenous insults.

Depending on the status of the HF growth cycle at the time of chemotherapeutic exposure, CIA may also manifest as telogen effluvium (TE) [104,105,106]. TE is typically induced by diverse acute or chronic psychophysiological stressors and is defined by the premature termination of anagen HFs and their synchronized transition into the telogen phase, resulting in excessive shedding of telogen hairs [73]. In severe cases of CIA, irreversible damage to HFSCs may occur, manifesting as inflammatory scarring alopecia [107].

In addition to direct cytotoxicity, various chemotherapy regimens have been reported to elevate the expression of proinflammatory cytokines, including IFN–γ, TNF-α, IL-1β, IL-2, IL-6, and IL-8 [108,109,110]. These inflammatory cytokines not only contribute to anti-tumor activity but also side effects, including hair loss, with inflammatory cell infiltration observed in HFs in CIA [111,112,113,114,115,116,117,118,119]. This convergence of DNA damage-induced stress and immune-mediated signaling reveals that CIA might be a multifaceted process, in which a wide array of stimuli share common pathways resulting in the failure of the HF immune-privileged state. The detailed molecular mechanisms of the pathogenesis of CIA and its effects on the HF microenvironment [9,10,120,121,122,123] will be elucidated in the following section.

### 3.1. p53-Mediated Apoptosis and Senescence: The Primary Upstream Signal

The rapid proliferative nature of anagen HF cells makes them exceptionally vulnerable to chemotherapeutic agents, which imposes a substantial DNA damage response (DDR) burden on the HF microenvironment. DNA damage is sensed by the sensory kinases ataxia telangiectasia and rad3-related (ATR) and ataxia telangiectasia mutated (ATM). These sensors subsequently phosphorylate and activate the effector kinases checkpoint kinase (CHK)-1 and CHK-2, respectively, which converge on p53 as the pivotal orchestrator of CIA [7,9,124]. Once induced, p53 coordinates follicular pathology through two distinct yet interrelated downstream cascades. The first involves p53-dependent apoptosis through reducing the B-cell lymphoma 2 (Bcl-2)/Bcl-2-associated X protein (Bax) ratio, coupled with augmentation of p53-upregulated modulator of apoptosis (PUMA) and Fas [12,120,124]. Fas signaling not only mediates HF keratinocyte and melanocyte apoptosis, but also stimulates the secretion of key inflammatory cytokines, including TNF-α and IL-15, alongside the aberrant expression of HLA [9,10,120,121,122,123]. Second, the p53-p21-p16 axis drives stress-induced premature senescence and cell cycle arrest, which is probably linked to HF cycle arrest [124].

Though HFSCs maintain their stemness through a relatively quiescent growth rate [125], which renders them less susceptible to chemotherapeutic toxicity that targets cell division mechanisms, excessive p53-related signaling or severe chemotherapy-induced mitotic defects can still induce the apoptotic loss of HFSCs, resulting in permanent CIA (pCIA) [7,126].

p53-mediated apoptosis also compromises vascular function by inducing vascular cell death, leading to tissue ischemia and alterations in vascular permeability. For instance, doxorubicin inhibits HF-associated angiogenesis while cyclophosphamide reduces vascular density and increases vascular permeability [127]. The reduced vascular density makes the lower portion of HFs, where cell division is more active during anagen, more susceptible to apoptosis [128]. Meanwhile, the increased vascular permeability enhances the penetration of chemotherapeutic agents into tissues, amplifying their cytotoxic effects on HFs.

While the p53-mediated axis constitutes a primary driver of CIA, clinical observations across diverse chemotherapeutic regimens and the varying efficacy of therapeutic agents indicate that the pathogenesis of CIA likely involves more complex signaling pathways [105]. The following sections will delineate how concurrent ROS and potential immune dysregulations act in concert with direct cytotoxicity to contribute to CIA.

### 3.2. ROS: Mediating Cytotoxicity and Potential Immune Dysregulation

Chemotherapy-induced ROS tend to aggravate cytotoxicity by damaging essential cellular components, including DNA, proteins, and lipids, thereby facilitating HF apoptosis [108,129,130,131] and premature catagen transition [121]. This relationship is exemplified by the fact that anthracyclines, cyclophosphamide, and platinum-based agents are the chemotherapeutic classes that generate the highest apoptotic levels of ROS; they simultaneously demonstrate the highest incidence of CIA [114,132,133].

Besides its cytotoxic impact, ROS can induce HF antigen-driven antibody production and trigger lipid peroxidation, both of which are thought to result in inflammation and HF-IP collapse [134,135,136,137]. If lipid metabolism is disrupted, the production of inflammatory cytokines in HFs, such as IL-6, will be amplified [65], thereby inhibiting hair bulb keratinocyte proliferation and interrupting the hair cycle [138].

The impact of ROS further extends to the hair bulge and affects HFSC clonal growth through the perturbation of epithelial–mesenchymal interactions and results in permanent hair loss [138,139]. Peroxisome proliferator-activated receptor gamma (PPAR-γ) agonists can neutralize ROS-induced lipid peroxidation to reverse pCIA [140].

### 3.3. IFN-γ: Linking Anti-Tumor Immunity to HF Damage

Chemotherapy-induced DDR leads to the accumulation of cytosolic DNA within both tumor cells and rapidly proliferating HF cells. This endogenous DNA can be sensed by the cyclic GMP-AMP synthase-stimulator of interferon gene (cGAS-STING) pathway, which can trigger IFN-γ signaling and a subsequent autoinflammatory response, a process characterized as immunogenic cell death (ICD) [15,141]. The release of cancer- or HF-derived damage-associated molecular patterns (DAMPs) such as self-DNA can be recognized by pattern recognition receptors (PRRs) like Toll-like receptor 4 (TLR4) and activate the cGAS-STING pathway in dendritic cells along with endoplasmic reticulum (ER) stress and ROS production [15,142,143]. These signals may activate the NOD-, LRR- and pyrin domain-containing protein 3 (NLRP3) inflammasome, promoting the increased expression of IL-1β and IFN-γ-producing CD8^+^ T cells [15,144]. IFN-γ has also been shown to enhance chemotherapy-induced cytotoxicity against tumor cells through the Janus Kinase-Signal Transducer and Activator of Transcription (JAK-STAT)-1 signaling pathway [145], which is linked to greater tumor control but heightened toxicity to hair bulb keratinocytes [46,146].

### 3.4. Psychophysiological Stress: The Mind-Skin-Hair Axis in CIA

CIA represents one of the most distressing side effects experienced by cancer patients [147], imposing not only physiological burdens but also profound psychological and social consequences [148,149]. This stress-driven pathway in hair loss disorders, such as AA, is characterized by neurogenic inflammation [73,150] and could be potentiated by ROS [151], which may also represent a secondary cause of hair loss that operates alongside direct chemotoxicity in CIA. Experimental murine models further demonstrate that sonic stress can induce HF apoptosis, suppress hair bulb keratinocyte proliferation, and increase perifollicular immune infiltration, including degranulated mast cells [152]. Additionally, both oxidative and emotional stress can disrupt melanogenesis and melanocyte function [153]. The physical manifestation of hair loss and the ensuing psychosocial impact of alopecia reciprocally reinforce each other, resulting in a vicious cycle.

Beyond its psychosocial ramifications, additional pathobiological and pharmacological evidence indicating that CIA may be underpinned by active inflammatory processes at follicular and tissue levels will be discussed below.

## 4. Additional Evidence of Postulated Immune Mechanisms Involved in CIA

### 4.1. Genetic Evidence

Genetic studies of CIA susceptibility reveal immune regulatory pathways in its pathogenesis. Genes encoding signal transducing adaptor molecule 2 (*STAM2*) are found to be correlated with CIA [154]. STAM2 is intrinsically linked to IL-2/JAK signaling pathways and plays a critical role in T cell development and immune function regulation [155,156]. CIA susceptibility has also been genetically associated with arachidonate 5-lipoxygenase activating protein (ALOX5AP), which is known to participate in inflammatory responses [157] and has been identified as a participant in the pathogenic pathways of immune-related scarring alopecia [158]. Complementary genetic analyses [116] have demonstrated potential associations between CIA and multiple immunologically relevant cytokines, including IFN-γ and IL-10, as well as T cell responses.

### 4.2. Clinical and Histopathological Evidence

Clinical observations provide evidence for the putative involvement of immune-mediated pathways in CIA. One case report [8] documented a compelling case of a patient without a prior history of AA who experienced recurrent hair loss five months after completing chemotherapy. The pattern of hair loss was highly selective, exclusively affecting pigmented hair, including brown eyebrows, eyelashes, axillary hair, and pubic hair, while sparing white scalp hair and skin. Additionally, the patient developed brittle fingernails and toenails, similar to the nail changes observed in AA.

The selective targeting of pigmented hair implies that chemotherapeutic agents may not only induce p53-mediated apoptosis but also subsequent antigen exposure and inflammation, rendering melanocyte-containing pigmented HFs particularly vulnerable to immune attack, which resembles autoimmune-mediated hair disorders. In contrast, the patient’s white hair appeared protected due to the absence of pigmentation. The observed pigment-specific pathology points toward the possible roles of melanocytes in CIA-associated immune dysregulation. Motl and Fausel [8] characterized this presentation as an “AA-like histopathology” and proposed that the patient’s cyclic hair loss pattern was associated with chemotherapy-related autoimmune alterations.

Another case series [159] conducted a detailed histopathological examination of ten patients with pCIA that lasted more than six months following taxane chemotherapy with adjuvant hormone therapy. All ten patients exhibited non-scarring alopecia with preserved HF unit structural integrity. Of note, two of those patients demonstrated peribulbar lymphoid cell infiltration around HFSCs, accompanied by pigment casts in HFs. Among those two cases, one patient presented without the clinical features of AA, displaying lymphoid cell infiltration in miniaturized HFs and responding favorably to minoxidil therapy. The second patient exhibited clinical manifestations consistent with AA, including persistent patchy alopecia, with lymphoid cell infiltration and pigment casts observed in diffuse regions of HFs, indicating pigment cell-associated IP collapse.

### 4.3. Trichoscopic and Histological Similarities Between CIA and AA

Trichoscopic examination of dystrophic anagen bulb hair in CIA further reveals numerous features that overlap with AA, including thin and scattered depigmented regrowing hair, yellow dots, black dots, broken hairs, exclamation mark hairs, Pohl-Pinkus constrictions, etc. [160,161,162,163,164].

The characteristic “flame hair” appearance in CIA, manifesting as irregular banding and variably pigmented short regrowing hairs [102,165], illustrates impaired hair pigmentation function. This dysfunction arises from chemotherapeutic toxicity targeting the follicular pigmentary unit. Consequently, aberrant pigment transfer occurs at ectopic sites within the hair bulb, leading to melanocyte dysregulation and impaired melanin transfer into keratinocytes [51,114], which mechanistically parallels immune-mediated processes observed in AA [71]. In addition to pigmentation defects, yellow and black dots reflect perifollicular infiltration combined with chemotherapy-induced cytotoxicity [165,166], and the diffuse presence of multiple yellow dots at trichoscopy could predict the severity of pCIA [167]. It has also been reported that patients with pre-existing damaged HFs and IP loss characteristic of AA demonstrate an increased susceptibility to chemotherapeutic toxicity [164].

### 4.4. Mechanistic Insights from Preventive and Restorative Therapeutic Interventions

Current strategies for managing CIA focus on either preventative measures that minimize initial cytotoxic insult or restorative treatments that address subsequent follicular dysfunction. Scalp cooling has received U.S. FDA approval and demonstrates high efficacy, particularly in patients receiving paclitaxel-only regimens, where hair retention rates as high as 100% have been reported [3]. The safety profile of scalp cooling is generally favorable, with common side effects limited to transient headaches and cold-induced discomfort. However, its use is typically contraindicated in patients with certain hematologic malignancies or cold-sensitivity disorders such as cryoglobulinemia [168,169].

For the management of established hair loss, minoxidil remains the primary pharmacological intervention for both CIA and pCIA [170,171]. Expanding upon its documented function in modulating ATP-sensitive potassium channels (KATP) in dermal papilla cells and enhancing follicular blood flow through vasodilatory properties, recent evidence suggests that minoxidil also exerts anti-inflammatory effects by reducing levels of IL-1α [172]. In the oncology population, low-dose oral minoxidil (0.5–5 mg/day) is considered generally safe and more effective for pCIA than the topical form [171]. Life-threatening complications such as pericardial effusion or Stevens-Johnson syndrome are exceedingly rare at these dosages [172]. More frequent and manageable side effects include transient hair shedding, hypertrichosis, headaches, orthostasis, and peripheral edema [172,173].

Consistent with these clinical priorities, a recent expert consensus reinforces that scalp cooling remains the cornerstone of CIA prevention during chemotherapy, while minoxidil is recommended as the primary restorative treatment for CIA and pCIA [4].

Beyond these established strategies for CIA, contemporary research has expanded to investigate pharmacological interventions that target immune-mediated mechanisms, reflecting an increasing recognition of the inflammatory components in CIA pathogenesis.

In the past, immunomodulatory agents have been shown to be beneficial in mitigating CIA severity. Cyclosporin and tacrolimus are found to antagonize the immune response by suppressing the production of IFN-γ, TNF-α, IL-17, and MHC class I molecules [34,44], thus inducing less severe HF injury affected by chemotoxicity [5,174,175]. Topical corticosteroids may also exert therapeutic effects in CIA by modulating immune-mediated pathways, preventing massive HF damage, particularly to HFSCs, thereby reducing the likelihood of pCIA [176].

Recent studies suggest that agents with immunomodulatory properties, including topical calcitriol and pentoxifylline, can attenuate chemotherapy-induced apoptosis of hair bulb keratinocytes [177,178]. These effects are thought to be mediated through the downregulation of IFN-γ, TNF-α, IL-2 receptor, and MHC class I molecules, along with the suppression of Th1-related immune responses [179,180,181,182,183].

Multiple agents bearing free radical scavenging capacities, such as keratinocyte growth factor (KGF), α-lipoic acid derivative, edaravone, and exogenous α-MSH, demonstrate efficacy in preventing or ameliorating CIA by counteracting genotoxic and oxidative stress in HFs and reducing the disruption of pigmentation [184,185,186,187].

**Figure 1:**
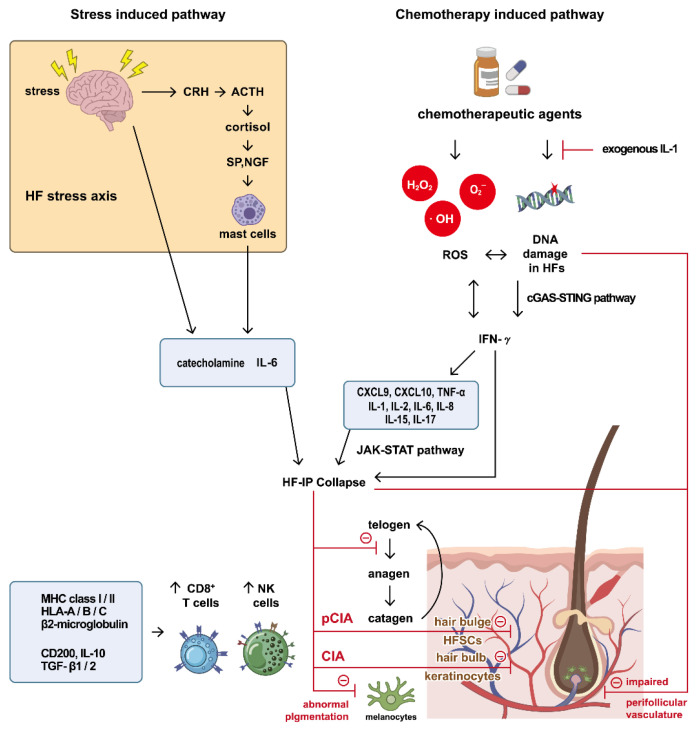
Proposed scheme of immune mechanisms involved in CIA. Arrows indicate illustrated interactions and literature-supported associations, but do not necessarily imply a direct, unidirectional causality in human CIA. (**Right**) Chemotherapy-induced pathway: chemotherapeutic agents induce DNA damage and ROS production in the HFs. DNA damage triggers the cGAS-STING pathway. Signaling cascades through DNA damage and ROS contribute to the upregulation of IFN-γ. The elevated IFN-γ level elicits the release of pro-inflammatory cytokines, including CXCL9, CXCL10, TNF-α, IL-1, IL-2, IL-6, IL-8, IL-15, and IL-17. The JAK-STAT pathway is a hypothesized key signaling mediator of these key inflammatory cytokines, which drives the activation and expansion of CD8^+^ T cells and NK cells and subsequent HF-IP collapse. Notably, exogenous IL-1 has been reported to exert a protective effect by retarding the hair cycle, thus shielding the HF from chemotoxicity that targets rapidly proliferating cells. (**Left**) Stress-induced pathway; psychological and physical stress (indicated by the lightning symbol) could activate the HF stress axis. This involves the release of CRH, ACTH, cortisol, SP, and NGF, which activate mast cells to release stress-induced catecholamines, which may further contribute to the inflammatory response by stimulating immune cells. (**Bottom**) The inflammatory milieu likely upregulates immunostimulatory markers (MHC class I/II, HLA-A/B/C, β2-microglobulin) and downregulates IP guardians (CD200, IL-10, TGF-β1/2), potentially contributing to HF-IP collapse and the subsequent inflammatory infiltration of CD8^+^ T cells and NK cells. HF-IP collapse disrupts the hair cycle by accelerating the anagen-to-catagen transition. The putative immune response targets: (1) rapidly proliferating hair bulb keratinocytes, causing acute (non-scarring) CIA; (2) antigenic melanocytes, leading to pigmentary changes; and (3) HFSCs at the bulge during severe inflammation, resulting in pCIA or scarring alopecia. Chemotherapy-induced cytotoxicity also impairs perifollicular vasculature, directly compromising hair growth. Abbreviations: ACTH, adrenocorticotropic hormone; CD8^+^, cytotoxic; cGAS-STING, cyclic GMP-AMP synthase-stimulator of interferon gene; CIA, chemotherapy-induced alopecia; CRH, corticotropin-releasing hormone; CXCL, C-X-C motif chemokine; HFs, hair follicles; HFSCs, hair follicle stem cells; HLA, human leukocyte antigen; IFN-γ, interferon-γ; IL, interleukin; IP, immune privilege; JAK-STAT, Janus Kinase-Signal Transducer and Activator of Transcription; MHC, major histocompatibility complex; NGF, nerve growth factor; NK, natural killer; pCIA, permanent CIA; ROS, reactive oxygen species; SP, substance P; TGF-β, transforming growth factor-β; TNF-α, tumor necrosis factor-α.

Taken together, genetic, histopathological, clinical, and therapeutic evidence points toward the possibility that CIA extends beyond simple cytotoxic injury to rapidly dividing hair bulb keratinocytes and instead reflects a broader process of complex secondary immune dysregulation (Figure 1). While the pathogenic mechanisms underlying CIA may not be entirely identical to those of AA (Table 1), immune responses likely maintain a non-negligible role in the pathogenesis of CIA. The observed overlap in the therapeutic targets between CIA and AA strengthens the rationale for exploring JAKi as an immunomodulatory approach to manage CIA. The next section discusses the details of corresponding therapeutic strategies utilizing JAKi for AA and CIA.

## 5. Recent Advances and Considerations for JAKi Therapy in AA and CIA

### 5.1. The JAK-STAT Signaling Pathway and Its Molecular Components in HF

The JAK-STAT signaling pathway serves two primary physiological functions: the regulation of immune responses, including the translation of proinflammatory cytokines, and the modulation of hematopoietic functions [207]. Each JAK isoform demonstrates specific receptor-binding preferences that determine its functional role in cytokine signaling. JAK1 is associated with the γ-chain receptor of various cytokines, including IL-6, IL-10, IL-13, and IFN-γ. JAK3 binds to the common IL-2 receptor γ-chain of the type I cytokine receptor group, mediating signals from IL-2, IL-4, and IL-15 [208,209]. While the specific role of JAK2 in this context remains to be fully elucidated [210], it is recognized as a key mediator of IFN-γ signaling along with JAK1.

JAK signaling plays a critical role in regulating the HF cycle by inducing telogen arrest, thereby preventing entry into the anagen phase. Conversely, inhibition of the JAK-STAT pathway has been demonstrated to promote the rapid onset of the anagen phase [211]. Aside from interfering with the hair cycle, the JAK signaling pathway also facilitates immune-mediated damage of hair bulb keratinocytes, HFSCs, and melanocytes. The JAK1/2-mediated phosphorylation of STAT1 triggered by IFN-γ results in the ectopic expression of MHC class I and II molecules, which subsequently leads to CD8^+^ T cell-mediated attack on HFs [212].

The recognition of the JAK-STAT pathway as a therapeutic target has led to the clinical application of various JAKi for managing hair loss disorders by suppressing proinflammatory cytokine signaling and restoring HF-IP. JAK inhibition reduces IFN-γ and IL-17 levels, leading to the downregulation of MHC class I and II molecules and CD8^+^ T cell-mediated immune responses [213,214,215] in the pathogenesis of AA [188,190,216]. Furthermore, JAKi have been shown to promote anagen entry through the modulation of vascular endothelial growth factor (VEGF) with a reported efficacy exceeding that of minoxidil in non-scarring alopecia [217].

### 5.2. Current Clinical Applications of JAKi in AA and Advances in cCIA

JAKi, including baricitinib, ritlecitinib, and deuruxolitinib, have received U.S. FDA approval for the treatment of AA. Other widely utilized off-label agents for hair loss disorders include tofacitinib and ruxolitinib [193,209]. All the above-mentioned JAKi demonstrate promising efficacy in promoting hair regrowth in AA, supported by multiple clinical trials and real-world evidence [193,194,218,219,220,221,222]. Clinical experience also suggests that patients who fail to respond to an initial JAKi may still achieve successful treatment results upon switching to a different agent [191]. Given the presumed immune-mediated features of CIA, the therapeutic potential of JAKi is increasingly explored in chemotherapy-induced models.

Guan et al. [17] conducted a study utilizing a cyclophosphamide-induced alopecia mouse model to evaluate the efficacy of tofacitinib encapsulated in a specialized nanoparticle carrier system. Their findings demonstrated that this nanoparticle formulation exhibited superior stratum corneum penetration and enhanced drug delivery capacity to HFs compared to conventional topical tofacitinib. Mechanistically, this formulation successfully antagonized the cyclophosphamide-induced upregulation of IFN-γ-mediated pathways, effectively suppressing the expression of MHC class I and II molecules and reducing CD8^+^ T cell infiltration [223]. Corroborating evidence reveals that the upregulation of genes within the JAK-STAT1 signaling pathway during chemotherapy is linked to the downregulation of Shh, a critical driver of the hair cycle, contributing to HF cycle arrest and subsequent hair loss [195]. Pharmacological inhibition of this axis via topical application of AG490 (a specific JAK2 inhibitor), ruxolitinib, or tofacitinib has been shown to counteract the suppression of Shh genes and mitigate follicular damage [195].

These findings indicate that JAKi may have therapeutic potential in CIA, acting through immunomodulatory mechanisms that resemble those observed in autoimmune AA. Building on these mechanistic insights and preclinical findings, the safety profiles and potential adverse effects of JAKi in patients with hair loss disorders warrant detailed examination.

### 5.3. Safety Stratification: Dermatological vs. Oncological Populations

A major limitation identified across clinical trials in hair loss disorders is that most JAKi fail to sustain long-term remission after treatment discontinuation, despite initially favorable responses [216,221,224]. This relapse phenomenon is thought to be driven by the persistence of pathogenic skin-resident memory T cells, which can rapidly reactivate immune responses upon re-exposure to HF autoantigens following JAKi withdrawal [210].

Given that sustained disease control often requires continuous JAKi administration, long-term safety profiles of JAKi demand nuanced interpretation based on the patient population. In dermatological cohorts, particularly in AA patients, these agents are generally well-tolerated. Common adverse events such as upper respiratory infections, headaches, acneiform eruptions, and dyslipidemia are typically mild and manageable [191,192,193,197,198,199,218,219,225,226].

Concerns regarding more serious complications, including major adverse cardiovascular events (MACE), malignancies, and thromboembolic events, primarily stem from observations in rheumatoid arthritis (RA) populations. However, current evidence indicates that these risks are highly dependent on baseline comorbidities and largely concentrated in high-risk subpopulations, such as patients over 65 years of age, long-term smokers, or individuals with pre-existing cardiovascular risk factors [227,228]. A recent propensity score-matched (PSM) analysis within RA populations found that tofacitinib and baricitinib did not significantly increase the risk of overall malignancy or skin cancer compared to biologic therapies [229]. Long-term data for baricitinib in RA spanning up to 9.3 years has shown stable rates of MACE, malignancies, and thromboembolic events [230], suggesting that the safety profile remains consistent over prolonged exposure [231]. It is also critical to consider that RA itself involves chronic systemic inflammation, which independently elevates malignancy risk, a factor less prevalent in most dermatological cohorts.

Safety data from AA cohorts exhibits a more favorable safety profile, as this younger population typically lacks the heavy inflammatory burden and multiple comorbidities found in RA [200,201]. Nevertheless, clinical vigilance, including periodic skin examination and monitoring, remains advisable when treating specific subpopulations, including patients with older age, a history of active or previous malignancy, significant cardiovascular comorbidities, and extensive exposure to multiple immunosuppressants [202], as some reports still highlight a potential risk of epithelial cancer, such as keratinocyte cancer associated with JAKi [203].

While JAKi show promising efficacy and an acceptable safety profile in AA, their application in CIA warrants particular scrutiny due to the unique vulnerability of this patient population. Patients undergoing chemotherapy frequently experience baseline hematologic toxicity and varying degrees of immunosuppression. The primary concern is that systemic JAK inhibition may aggravate anemia or thrombocytopenia and interfere with NK cell-mediated anti-tumor immunity [204,205,209,232]. Recent expert consensus emphasizes that while JAKi can be considered for cancer patients, their use necessitates a thorough multidisciplinary assessment in collaboration with oncologists to balance between the therapeutic goal of hair restoration in CIA and the priorities of oncological safety [201,206].

### 5.4. Innovation in Topical Formulations and Delivery Systems

Given the potential risks, topical JAKi formulations represent an attractive, safer alternative for high-risk patients or individuals with localized disease [192,209,211,222,233]. The adverse effects of topical JAKi are primarily localized skin irritation, itching, and acneiform or rosacea-like skin changes [234]. However, the clinical efficacy of traditional topical formulations has been proven to be less effective than oral counterparts, likely due to limited skin penetration and suboptimal delivery to the HFs [192,222].

To address these limitations, innovative topical delivery systems are being explored, and the variable success of localized therapy is often attributed to the vehicle used. For example, case reports have documented complete hair regrowth with 2% tofacitinib in a liposomal base after a total lack of response to the same concentration in a conventional VersaBase cream [235,236]. Further innovation involves nanoparticle carrier systems [17] or microneedling-assisted delivery strategies to overcome the skin barrier [237]. Such localized approaches could enhance intrafollicular bioavailability while bypassing the systemic toxicities that typically preclude oral JAKi use in oncological patients. These innovative delivery systems represent a promising therapeutic avenue for managing CIA, offering a safer alternative to integrate JAKi into the supportive care of cancer survivors.

## 6. Future Research Directions: The Dual Roles of IL-15 and IL-1 in Hair Disorders

### 6.1. Compartment-Specific Duality of IL-15 and Therapeutic Considerations in Hair Disorders

IL-15 likely exhibits as a complex, site-dependent mediator in JAKi therapy in autoimmune hair disorders. Accumulating evidence has indicated that systemic and intrafollicular IL-15 signaling can exert fundamentally distinct and even opposing biological effects, necessitating careful therapeutic consideration.

#### 6.1.1. Pathogenic Role of Systemic IL-15 in HF-IP Collapse

At the systemic level, IL-15 functions as a pro-inflammatory cytokine. Elevated levels of circulating IL-15 induced by IFN-γ and TNF-α can amplify inflammatory cascades in AA [214,238,239,240], thereby promoting CD8^+^ cell cytotoxicity and supporting the survival of pathogenic memory T cells [18,241,242]. This sustained immune activation drives the collapse of HF-IP, and JAKi effectively suppresses IL-15–driven extrafollicular pathogenic axis, resulting in attenuation of autoimmune-related hair loss [194].

#### 6.1.2. Hair-Growth-Protective and Promoting Effects of Intrafollicular IL-15

In contrast to its systemic effects, IL-15 signaling within the HF microenvironment appears to preserve IP integrity by maintaining the expression of insulin-like growth factor-1 (IGF-1), α-MSH, and TGF-β, while counteracting IFN-γ–induced inflammation. At appropriate concentrations, recombinant human IL-15 (rhIL-15) promotes hair growth by prolonging the anagen phase, delaying catagen onset, increasing proliferative keratinocyte populations, and reducing apoptosis within hair bulbs [243]. Experimental evidence shows that impaired IL-15Rα signaling leads to the upregulation of MHC class I molecules and β2-microglobulin, highlighting the critical role of IL-15 in sustaining HF-IP [243,244]. IL-15 agonism has been shown to inhibit the apoptosis of hair bulb keratinocytes, supporting its potential therapeutic application in CIA [245].

The compartment-specific duality of IL-15 signaling constitutes a clinically relevant paradox. While systemic JAK inhibition effectively suppresses pathogenic extrafollicular IL-15 signaling, it may simultaneously interfere with protective intrafollicular IL-15Rα-mediated pathways, potentially increasing susceptibility to disease relapse once treatment is discontinued. This underscores the need for compartment-selective therapeutic strategies. Targeted activation of intrafollicular IL-15 signaling, potentially via topical or follicle-restricted approaches, may restore IP and promote hair regeneration, while minimizing systemic immune activation and adverse effects.

### 6.2. Context-Dependent Roles of IL-1 in Hair Disorders

The pathogenic role of IL-1 in hair loss disorders appears to be governed not merely by its concentration, but also by the temporal dynamics and the cellular source of its induction.

#### 6.2.1. Pathogenic Role of IL-1 in HF-IP Collapse

Under physiological and inflammatory conditions, such as AA, IL-1α, and IL-1β, are well established as potent inhibitors of HF growth [189]. Exposure to IL-1 induces dystrophic anagen HFs and perifollicular inflammation, contributing to HF-IP collapse, keratinocyte apoptosis [46,54,246,247,248] and tissue damage [42,95,196,197,198]. In this context, endogenous IL-1 production is a hallmark of tissue damage and disease progression.

#### 6.2.2. Timing Dependent Protective Role of IL-1 in CIA

IL-1 exhibits a context- and timing-dependent protective role in CIA when administered exogenously prior to cytotoxic insult. IL-1 is thought to induce a transient, low-proliferative or regressive state in the HFs, thereby reducing the vulnerability of rapidly cycling hair bulb keratinocytes to chemotherapy-induced cytotoxicity [249]. Immunomodulators such as ImuVert and AS101 have been shown to prevent cytarabine-induced alopecia in murine models and to attenuate CIA severity in patients receiving carboplatin- or etoposide-based chemotherapy, effects that have been associated with transient elevations of IL-1α and IL-1β [250,251,252]. The direct administration of IL-1 has also demonstrated a protective efficacy in CIA models [253]. Notably, endogenous IL-1 triggered during or after chemotherapy remains purely pathogenic. Chemotherapy itself triggers inflammatory cascades that elevate IL-1β, which predominantly acts as a tissue-damaging cytokine and exacerbates follicular injury [254]. These observations underscore the hypothesis that the beneficial effects of IL-1 in CIA are highly dependent on exogenous delivery, precise timing, and local context, rather than uncontrolled inflammation-driven IL-1 production.

Collectively, these findings highlight the complexity of cytokine-mediated regulation of HF homeostasis and injury responses. Future studies should further delineate the context-specific roles of IL-15 and IL-1 in the pathogenesis of CIA, enabling the development of refined therapeutic strategies.

## 7. Conclusions

This comprehensive review establishes that CIA may be a multifaceted disorder that extends beyond direct cytotoxic injury to follicular structures. Converging evidence from genetic, histopathological, clinical, and therapeutic studies indicates substantial immune-mediated involvement analogous to autoimmune AA, including inflammatory cell infiltration and cytokine-driven follicular damage and IP collapse. Overall, CIA reflects the combined effects of chemotherapy-induced DNA damage and stress-amplified neuroimmune dysregulation.

Beyond the many immunomodulatory agents previously used in CIA, JAKi therapy appears to act primarily through immunosuppressive mechanisms. However, emerging research reveals that JAKi may also exert hair-intrinsic effects independent of lymphocyte activity, including the modulation of Shh signaling pathways, the upregulation of growth factors such as VEGF, and the direct stimulation of melanogenesis [195,211,214,255]. Whether the therapeutic efficacy of JAKi in CIA derives primarily from immunomodulatory or broader hair growth-promoting effects warrants additional studies.

While JAKi demonstrate favorable safety profiles in dermatological AA populations, their application in cancer patients requires additional considerations. JAKi may exacerbate chemotherapy-related toxicities, impair intrinsic anti-tumor activity, and demonstrate associations with skin malignancies [203,204]. Even topical administration requires evaluation, given cutaneous metastasis risks [256]. Maintaining adequate immune surveillance while treating alopecia represents a critical challenge, necessitating careful patient selection, comprehensive baseline assessments, and rigorous monitoring protocols.

The integration of insights from autoimmune alopecia research with CIA-specific investigations reveals that CIA likely involves inflammatory components and requires novel therapeutic strategies. Additionally, the context-dependent roles of key cytokines, including IL-1 and IL-15, underscore the necessity for individualized treatment protocols. The challenge lies in determining how anti-inflammatory interventions should be calibrated without suppressing critical inflammatory pathways that protect against hair loss or compromising cancer treatment efficacy and patient safety. Future investigations are needed to prioritize targeted delivery systems, dosing optimization, and comprehensive long-term safety assessments to establish evidence-based guidelines. CIA may ultimately be transformed into a preventable or treatable condition, improving the quality of life for millions of cancer patients worldwide.

## Figures and Tables

**Table 1:** Comparative pathogenic mechanisms and therapeutic considerations in AA and in CIA.

	AA	CIA
Triggering Factors	Multifactorial, mainly genetic predisposition with environmental triggers, including psychological or physiological stress [85,188,189]	DNA damage and ROS production in rapidly proliferating HFs by direct chemotoxicity [6,9,12,131]
Proposed Key Immune Pathways/Cytokines	Th1-mediated inflammation and HF IP collapse induced by IFN-γ, IL-2, IL-15, etc. [13,14,29,37,38,39]	p53 signaling induced apoptosis, senescence, and programmed cell death in HFs and HFSCs [7,12,121,124] Potential HF-IP collapse caused by inflammatory cytokines, including IFN-γ, TNF-α, IL-2, and IL-15 as downstream mediators of DDR through the cGAS-STING pathway, ROS [15,112,113,116,130,143,154], and follicular stress axis from psychological stress [16,147,148,149,151,153]
Shared Features of HF Pathology	Perifollicular lymphocytic infiltrates with pigment casts, yellow dots, black dots, broken hairs, short vellus hairs, exclamation mark hairs, Pohl-Pinkus constrictions, hypopigmented or hyperpigmented regrowing hairs (known as flame hair in CIA), and diffuse erythema [71,102,107,160,161,162,163,164,165,166,167]	
Therapeutic Approaches	Mainly focusing on immunosuppressive or immunomodulatory agents such as steroids or JAKi in severe cases [190,191,192,193,194], with hair growth stimulants such as minoxidil [172]	Most CIA resolves spontaneously within weeks to months after cessation of cytotoxic therapy Most effective preventive strategy during chemotherapy exposure: scalp cooling [2,3,4] For established CIA: hair growth stimulants such as minoxidil [4,170,171,173] Miscellaneous immunomodulatory agents [1,5,17,174,175,176,177,178,179,180,181,182,183], including JAKi [17,195], bear the potential to prevent or treat the underlying inflammatory process and associated HF-IP collapse proposed in CIA
Autoantibody Association	Genetic predisposition of autoimmunity for self-antigens, triggered by intrinsic or extrinsic stressors [85,188,189]	Putative tissue damage induced massive self-antigen exposure and inflammation-associated autoimmunity [15,70,103,110,142,144]
Role of IFN-γ	Closely related to IFN-γ-induced Th1-mediated autoimmune response, with serum IFN-γ associated with AA severity and commonly used as a trigger in experimental AA models [37,38,196]	IFN-γ, along with ROS, TNF-α, IL-2, IL-15 are essential in anti-tumor activity but may simultaneously cause Th1 cell attack to HFs without priming antigen presentation [46,111,116,118,146,154]
Safety Considerations for JAKi Use	Generally well-tolerated in younger dermatological cohorts with mild adverse effects (headache, acne, dyslipidemia, etc.) [191,192,197,198,199], but caution is needed for elderly or those with cardiovascular/malignancy history [200,201,202] Standard monitoring including baseline and periodic CBC, lipid profile, and skin examinations [203,204]	Lacking clinical trials including patients with a history of malignancy Vigilant monitoring requiring collaboration with oncologists regarding the risks of baseline hematologic toxicities exacerbation or interference with NK-cell-medicated anti-tumor immunity [205,206]

Abbreviations: AA, alopecia areata; CBC, complete blood count; CD8^+^, cytotoxic; cGAS-STING, cyclic GMP-AMP synthase-stimulator of interferon gene; CIA, chemotherapy-induced alopecia; DDR, DNA damage response; HFs, hair follicles; HFSCs, hair follicle stem cells; IFN-γ, interferon-γ; IL, interleukin; IP, immune privilege; JAKi, Janus kinase inhibitor; NK, natural killer; ROS, reactive oxygen species; Th1, Type 1 T helper; TNF-α, tumor necrosis factor-α.

## Data Availability

No new data were created or analyzed in this study. Data sharing is not applicable to this article.

## Abbreviations

The following abbreviations are used in this manuscript:

AA	alopecia areata
ACTH	adrenocorticotropic hormone
ALOX5AP	arachidonate 5-lipoxygenase activating protein
APC	antigen-presenting cell
ATM	ataxia telangiectasia mutated
ATR	ataxia telangiectasia and rad3-related
Bax	Bcl-2-associated X protein
Bcl-2	B-cell lymphoma 2
CBC	complete blood count
CD8^+^	cytotoxic
CHK	checkpoint kinase
cGAS-STING	cyclic GMP-AMP synthase-stimulator of the interferon gene
CGRP	calcitonin gene-related peptide
CIA	chemotherapy-induced alopecia
CRH	corticotropin-releasing hormone
CXCL	C-X-C motif chemokine
DAMP	damage-associated molecular pattern
ER	endoplasmic reticulum
HF	hair follicle
HF-IP	hair follicle immune privilege
HFSC	hair follicle stem cell
HLA	human leukocyte antigen
HPA	hypothalamic–pituitary–adrenal
IP	immune privilege
ICD	immunogenic cell death
IDO	indoleamine 2,3-dioxygenase
IFN-γ	interferon-γ
IGF-1	insulin-like growth factor-1
IL	interleukin
IL-15Rα	IL-15 receptor-α
JAK	Janus kinase
JAKi	Janus kinase inhibitor
JAK-STAT	Janus Kinase-Signal Transducer and Activator of Transcription
MACE	major adverse cardiovascular events
MHC	major histocompatibility complex
MIF	migration inhibitory factor
α-MSH	α-melanocyte-stimulating hormone
NGF	nerve growth factor
NK	natural killer
NK-1	neurokinin-1
NLRP3	NOD-, LRR- and pyrin domain-containing protein 3
pCIA	permanent CIA
POMC	pro-opiomelanocortin
PPAR-γ	peroxisome proliferator-activated receptor gamma
PRR	pattern recognition receptor
PSM	propensity score-matched
PUMA	p53-upregulated modulator of apoptosis
RA	rheumatoid arthritis
rhIL-15	recombinant human IL-15
ROS	reactive oxygen species
TLR4	Toll-like receptor 4
SCF	stem cell factor
Shh	Sonic hedgehog
SP	substance P
TE	telogen effluvium
TGF-β	transforming growth factor-β
Th1	Type 1 T helper
TNF-α	tumor necrosis factor-α
VEGF	vascular endothelial growth factor
